# Systemic Immune Inflammatory Index as Predictor of Blood Pressure Variability in Newly Diagnosed Hypertensive Adults Aged 18–75

**DOI:** 10.3390/jcm13226647

**Published:** 2024-11-06

**Authors:** Yücel Karaca, Mehdi Karasu, Mehmet Ali Gelen, Şeyda Şahin, Özkan Yavçin, İrfan Yaman, Şıho Hidayet

**Affiliations:** 1 Department of Cardiology, Fethi Sekin Sehir Hastanesi, 23280 Elazıg, Turkey; yucel.karaca1@saglik.gov.tr (Y.K.); maligelen4723@gmail.com (M.A.G.); seydshn.58@gmail.com (Ş.Ş.); ozkanycn@hotmail.com (Ö.Y.); 2 Department of Cardiology, Gaziantep Şehir Hastanesi, 27470 Gaziantep, Turkey; irfanyaman89@gmail.com; 3 Department of Cardiology, Malatya İnönü Üniversitesi Tıp Fakültesi, 44280 Malatya, Turkey; shhidayet@hotmail.com

**Keywords:** systemic immune inflammatory index, ambulatory blood pressure variability, hypertension

## Abstract

**Background:** Accumulating evidence from clinical trials, large registries, and meta-analyses of population studies shows that increased Blood Pressure Variability (BPV) is predictive of Cardiovascular (CV) outcomes, independently of the average Blood Pressure (BP) values. One of the mechanisms explaining the relationship between BPV and target organ damage is the inflammatory response. The Systemic Immune Inflammation Index (SII), which relies on peripheral blood cell counts, including platelets, neutrophils, and lymphocytes, has emerged as a predictor of prognosis and outcomes in various diseases. The aim of this study was to investigate the association of the SII with Ambulatory Blood Pressure Variability (ABPV) in newly diagnosed hypertensive patients. **Methods:** This study was designed as a cross-sectional observational study. A total of 1606 consecutive newly diagnosed Hypertension (HT) patients were included in the study. The population was evaluated across 3 different categories according to HT grades (5 groups), eligibility for antihypertensive therapy (2 groups) and ABPV levels (2 groups). **Results:** Significant differences were observed between ABPV groups in terms of Neutrophil to Lymphocyte ratio, Platelet to Lymphocyte ratio, glucose, SII, high-sensitive CRP, HT grade, Inter-Ventricular Septum, Posterior Wall thickness, and Left Ventricular Mass (*p* < 0.005). There was a significant relationship between SII and ABPV (r: 0.619, *p* < 0.05). At the cutoff value of 580.49, SII had 77% sensitivity and 71% specificity for ABPV > 14 (AUC: 0.788). **Conclusions:** SII may assist in developing an early treatment approach to minimize complications in patients with high ABPV who are at a higher risk of CV events.

## 1. Introduction

Hypertension (HT) stands as one of the foremost preventable cardiovascular risk factors [1]. It is well established that hypertensive individuals face a higher risk of cardiovascular (CV) mortality, cerebrovascular diseases, renal failure, and retinopathy compared to their normotensive counterparts [2,3,4].

In daily life, blood pressure (BP) values fluctuate due to various activities, and these fluctuations can have significant implications for health. To provide a more representative estimate of BP levels than traditional clinical measurements, 24 h Ambulatory Blood Pressure Monitoring (ABPM) has emerged as a valuable tool [5]. ABPM not only offers a more accurate reflection of BP but is also closely associated with target organ damage, surpassing the predictive power of office BP measurements [5].

Extending Ambulatory Blood Pressure Monitoring (ABPM) to 48 h or beyond could indeed provide a more comprehensive assessment of Blood Pressure Variability (BPV), potentially capturing a fuller range of daily activities and sleep patterns. Studies indicate that prolonged ABPM may offer better prognostic accuracy for cardiovascular outcomes, particularly in patients with suspected high variability [6,7]. The additional data could also improve the reliability of BP variability indices, further clarifying the relationship between BP variability and inflammatory markers like SII.

However, there are some feasibility challenges. Longer monitoring periods could reduce patient compliance due to discomfort, interruptions to daily routines, or issues with the device, which could lower data quality if patients alter their behaviors. Additionally, ABPM devices are calibrated for 24 h intervals, so extending to 48 h might require adjustments in calibration and data interpretation.

Individuals with greater BP variability may experience more pronounced target organ damage than those with a stable 24 h mean BP [8]. Metrics like the standard deviation (SD) of 24 h BP, which quantifies BP variability, have been shown to correlate with the progression of organ damage over time [9].

Furthermore, despite the diagnostic challenges posed by BP variability, accumulating evidence from clinical trials, large registries, and population-based meta-analyses underscores the prognostic significance of Blood Pressure Variability (BPV) in predicting CV outcomes, independently of average BP values [10,11,12,13,14]. Moreover, BPV appears to extend its implications beyond CV outcomes, affecting a broader spectrum of disease outcomes and complications [15,16,17].

Inflammation has been well recognized as a contributor to the pathophysiology of HT [18]. Among the mechanisms connecting BPV to target organ damage, the inflammatory response has garnered attention. Experimental evidence suggests that elevated BP and BPV may stimulate endothelial expression of cytokines and promote inflammation [19]. However, the precise relationship between BPV and inflammation in individuals with HT remains unexplored.

The Systemic Immune Inflammation Index (SII), which relies on peripheral blood cell counts, including platelets, neutrophils, and lymphocytes, has emerged as a predictor of prognosis and outcomes in cancer and cardiac patients [20,21,22]. A previous study demonstrated SII as an independent predictor of major adverse CV events in coronary artery disease (CAD) patients [21]. Although an association between non-dipper HT, which was characterized by a drop of less than 10% in systolic and diastolic BP, and SII has been noted in a prior study [23], its relationship with variability indices and HT grades has yet to be established.

Hypertension (HT) is one of the foremost preventable cardiovascular risk factors, particularly affecting specific demographics with age- and health-based susceptibilities. In our study, we focused on newly diagnosed hypertensive patients within a defined age range and without significant comorbidities to understand the role of ambulatory blood pressure variability (ABPV) in predicting outcomes for this demographic.

With these considerations in mind, we designed this study to investigate the association of SII with Ambulatory Blood Pressure Variability (ABPV) in newly diagnosed hypertensive patients. This investigation aims to shed light on the interplay between SII, BPV, and HT severity, potentially informing early interventions to mitigate complications in hypertensive individuals at higher CV risk.

## 2. Methods

This cross-sectional observational study included newly diagnosed hypertensive patients aged 18–75, who were otherwise free from severe comorbid conditions such as coronary artery disease, renal impairment, or peripheral artery disease. Patients meeting these criteria were selected to specifically address the predictive value of the Systemic Immune Inflammation Index (SII) in an age- and health-defined population, ensuring the study’s relevance to this subset of the hypertensive population. It was conducted in accordance with the International Code of Ethics and the Declaration of Helsinki, and it received approval from the local ethics committee (approval number: 2022/06, date: 26 January 2022). Informed written consent was obtained from all of the participants prior to the study.

The study included a total of 1606 consecutive newly diagnosed HT patients who presented to the cardiology outpatient clinic of İnönü University between 1 June 2022 and 1 April 2023, either with complaints of high blood pressure (BP) or who were found to have high BP without any complaints. To ensure that the study included patients with pure hypertension, the exclusion criteria were broad. Individuals under 18 years of age or over 75 years of age, those with secondary HT, known coronary artery disease (CAD), peripheral artery disease, heart failure, arrhythmias, significant valvular diseases, shift workers, estimated glomerular filtration rate (eGFR) < 90 mL/Minute/1.73 m^2^ (indicating renal failure), and those using antihypertensive drugs, catecholamines, α- or β-blockers, or tranquilizers were excluded. A total of 1220 patients were excluded from the study based on these criteria, leaving data from the study group, consisting of 386 patients, for analysis.

At the time of their initial visit, patient data, including age, gender, echocardiographic information, and laboratory results, were collected. Blood samples were drawn between 8 and 10 am following a 12 h fasting period to assess hemogram, biochemistry, and high-sensitivity C-Reactive Protein (hs-CRP) levels. The Systemic Immune Inflammation Index (SII) was calculated using the formula Platelet × Neutrophil/Lymphocyte (P × N)/L, where P, N, and L denote platelet, neutrophil, and lymphocyte counts in peripheral blood, respectively [23]. Body mass index (BMI) was calculated.

Office BP measurements were taken with an aneroid sphygmomanometer using a cuff that measured between 36 and 42 cm in width (ERKA Perfect-Aneroid; ERKA, BadTölz, Germany). Two measurements were obtained after a 5 min rest, and the average was recorded. Patients with a BP reading of 140/90 mmHg or higher were referred for Ambulatory Blood Pressure Monitoring (ABPM).

A fully automatic BP monitoring device (BR-102 Plus, Schiller, Baar, Switzerland) was used for 24 h ABPM. The non-dominant arm was selected for cuff placement. Measurements were taken every 10 min during the day and every 20 min at night. The device was reinserted in patients with less than a 70% measurement completion rate. Awake and sleep times were determined based on patient-provided information. Nighttime BP reduction (percent) was calculated using the following formula: 100 × [1 − (sleep systolic BP/awake systolic BP)] [23]. Dipping status was defined as a decline in systolic and diastolic BP of greater than 10%, while non-dipper HT was characterized by a drop of less than 10%. BP variability was quantified using the standard deviation (SD) of systolic blood pressure (SBP) as an index [2,24].

Based on the ABPM results, patients were categorized according to BP measurements following the American Heart Association (AHA) 2017 guideline [25] (Table 1).

Normal: SBP less than 120 and DBP less than 80 mm Hg. High–normal: SBP 120 to 129 and DBP less than 80 mm Hg. Stage 1 hypertension: SBP 130 to 139 or DBP 80 to 89 mm Hg. Stage 2 hypertension: SBP greater than or equal to 140 mm Hg or DBP greater than or equal to 90 mm Hg. Severe hypertension defined as SBP ≥ 180 mmHg or DBP ≥ 120 mmHg.

To provide a comprehensive overview of the patient population’s clinical, laboratory, and demographic characteristics, the population was analyzed across three different categories:

1. Category 1: The population was divided into 5 groups (Group 1: normal BP, Group 2: high-normal BP, Group 3: grade 1 HT, Group 4: grade 2 HT, Group 5: severe HT) based on ABP values.
2. Category 2: The population was divided into two groups (Group 1: normal BP, high-normal BP, and grade 1 HT; Group 2: grade 2 and severe HT) based on eligibility for starting antihypertensive therapy.
3. Category 3: After calculating ABPV, the fuzzy *c*-means (FCM) algorithm [26] was applied to divide patients into low–intermediate (Group 1: ABPV ≤ 14) and high (Group 2: ABPV > 14) variability groups. Employing a clustering algorithm instead of dividing patients according to percentiles was preferred, because theoretically this approach is able to establish optimal boundaries, which ensure that individuals placed in the same class are the most similar and separated ones are the most dissimilar.

Echocardiography was performed by an experienced cardiologist who was blinded to the patient clinical and laboratory data using the Vivid 9 (General Electric Healthcare, Chicago, IL, USA) system with a 2.5–3.5 MHz transducer. The examination was conducted with patients in the left decubitus position, measuring the interventricular septum (IVS) and posterior wall (PW) thicknesses. The left ventricular mass index (LVMI) was calculated by dividing left ventricular mass (LVM) by body surface area. All measurements were repeated at least three times and averaged.

## 3. Statistical Analysis

Statistical analyses were conducted using the Statistical Package for the Social Sciences (SPSS Inc., Chicago, IL, USA) for Windows version 21.0. The normality of continuous variables was assessed using the Kolmogorov–Smirnov test. Normally distributed variables are presented as mean ± standard deviation, while non-normally distributed variables are presented as median (minimum–maximum) values. Descriptive statistics include percentages and absolute values.

To compare baseline clinical characteristics among the five groups, we employed an analysis of variance (ANOVA) test. Due to the inability to achieve a normal distribution in the ANOVA test conducted for the hypertension groups and a notable difference in group sizes, the Games–Howell test was employed for post hoc analysis. Given the presence of five distinct groups, we applied the Bonferroni correction to mitigate the margin of error.

Sociodemographic and clinical characteristics of two groups were compared using independent sample t-tests. Logistic regression models were utilized to determine whether SII was independently associated with grade 2 and severe hypertension.

Receiver operating characteristic (ROC) analysis was performed to identify the most sensitive SII cutoff level for identifying grade 2 and severe hypertensive patients.

Correlation analysis was employed to investigate the relationship between Ambulatory Blood Pressure Variability (ABPV) and SII.

Additionally, linear regression analysis was conducted to determine the effects of independent variables on ABPV.

Finally, ROC analysis was used to determine the most sensitive SII cutoff level for identifying patients with ABPV > 14.

## 4. Results

**Figure 1:**
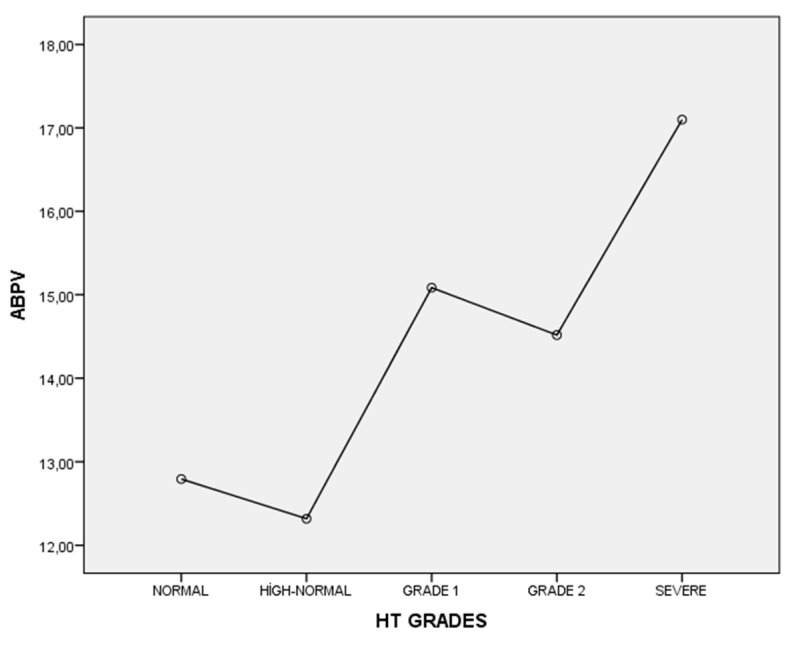
Mean levels of ABPV according to hypertension grades.

Table 1 outlines the general demographic and clinical characteristics of the hypertension groups, categorized by HT grades. Multiple comparisons were conducted, and detailed differences between the groups are presented in both Table 1 and Figure 1.

The ABPV value was significantly lower in the normal blood pressure group compared to the severe hypertension group (*p* < 0.001). The ABPV value was significantly lower in the high–normal blood pressure group compared to the grade 1, grade 2, and severe hypertension groups (*p* < 0.005). In the grade 2 hypertension group, the ABPV value was significantly higher than the high–normal blood pressure group but significantly lower than the severe hypertension group (*p* = 0.002). In the severe hypertension group, the ABPV value was significantly higher compared to the normal blood pressure, high-normal blood pressure, and grade 2 hypertension groups (*p* < 0.005).

Table 2 demonstrates a significant difference between the two BP groups categorized by eligibility for antihypertensive therapy in terms of several variables, including SII, neutrophil to lymphocyte ratio (N/L), platelet to lymphocyte ratio (P/L), White Blood Cell count (WBC), neutrophil count (N), lymphocyte count (L), platelet count (Plt), Ambulatory Blood Pressure Variability (ABPV), and the presence of a dipping pattern (*p* < 0.05). Notably, gender showed a significant difference when comparing five different groups based on HT grades, but no significant difference was observed in the two-group comparison based on eligibility for antihypertensive therapy.

**Figure 2:**
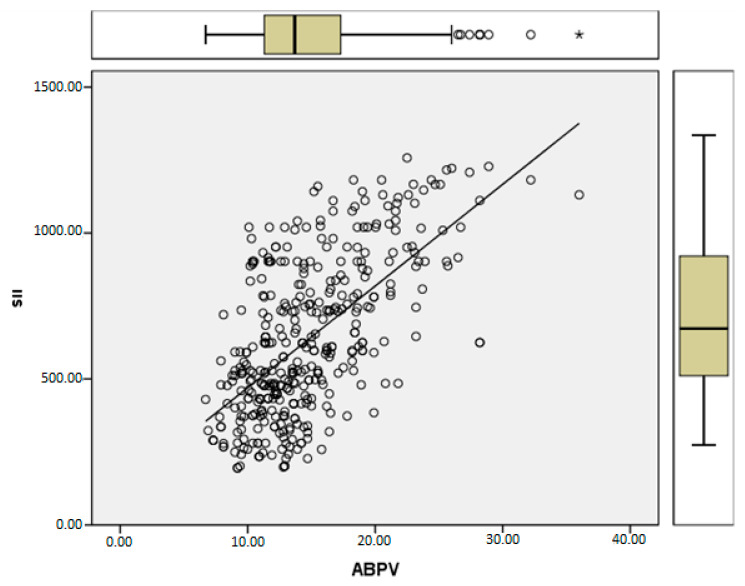
Correlation between SII and ABPV. SII: mean ± Std: 635.45 ± 265.17; ABPV: mean ± Std: 14.75 ± 4.71; Pearson Correlation: 0.619; *p*: = 0.000; * correlation is significant at the 0.01 level.

Table 3 reveals significant differences between ABPV groups in terms of N/L, P/L, glucose, Left Ventricular Mass (LVM), SII, high-sensitivity C-Reactive Protein (hs-CRP), HT grade, Posterior Wall thickness (PW), and Interventricular Septum thickness (IVS) (*p* < 0.005). Specifically, N/L, P/L, glucose, LVM, SII, hs-CRP, HT grade, PW, and IVS were found to be higher in the ABPV > 14 group, while no significant difference was observed between the groups concerning gender. Correlation analysis indicated a significant relationship between SII and ABPV (Figure 2) (Pearson Correlation: 0.619, *p* < 0.05).

**Figure 3:**
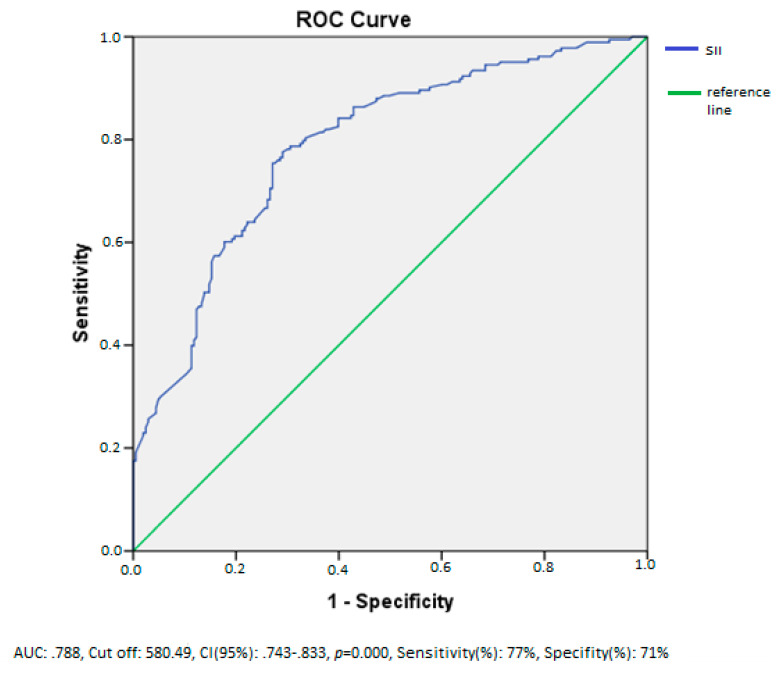
ROC analysis of SII performed to predict ABPV > 14.

After conducting simple linear and multiple linear regression analyses to assess the impact of independent variables on ABPV, only SII was identified as an independent predictor of ABPV (Table 4). Due to multicollinearity, WBC, N, L, and Plt were not included in the regression analysis. According to the ROC analysis results, SII exhibited a sensitivity of 77% and specificity of 71% for identifying ABPV > 14 at a cutoff value of 580.49 (AUC: 0.788) (Figure 3).

Furthermore, there was a statistically significant difference between the dipper and non-dipper groups in terms of SII and ABPV (*p* < 0.05). However, there was no significant difference between the ABPV groups concerning dipper status.

In examining SII’s predictive value for ABPV, the results are most applicable to patients fitting the study’s demographic and health profile, as our inclusion criteria excluded patients with significant health conditions or those outside the defined age range. This ensures a more targeted application of the findings within a specific hypertensive patient population.

## 5. Discussion

The primary finding of our study underscores a notable correlation between the Systemic Immune Inflammatory Index (SII) and Ambulatory Blood Pressure Variability (ABPV), further identifying SII as an independent predictor for ABPV > 14.

Previous reports have also demonstrated significant associations between inflammatory markers and elevated blood pressure (BP) in seemingly healthy individuals [19]. Moreover, the link between high-sensitivity C-reactive protein (hs-CRP) levels and the development of hypertension (HT) indicates the inflammatory component of HT [27]. Kawada et al. have established an independent relationship between neutrophil levels and HT [28]. Platelet aggregation tendencies are heightened in hypertensive patients [29], as reported in our study, which showed a significant increase in SII levels among patients with grade 2 and severe HT.

Individuals with non-dipper HT exhibit a more pronounced inflammatory response [30]. A previous study by Akyüz et al. found that SII was elevated in the non-dipper HT group and served as an independent indicator of non-dipper HT [23]. In our study, although SII was significantly higher in the ABPV > 14 group, there was no difference between the two groups in terms of dipper status.

In our study, we showed that increased HT grades have increased ABPV values. Although there were no statistically significant differences between groups, the normal HT group had higher ABPV levels compared to the high–normal HT group and grade 1 patients had higher ABPV levels than grade 2 patients. This observation could be explained by several factors:

Physiological Compensation [31]: Individuals in the higher grades may have early regulatory adaptations, such as enhanced vascular responsiveness or autonomic adjustments, that buffer BP fluctuations more effectively. This could lead to relatively stable BP readings despite a trend toward higher baseline BP.

Early Hypertensive Changes Without Variability Increase [32]: In some cases, as BP levels gradually increase toward hypertensive ranges, variability may initially remain low. This could indicate that in early stages of BP elevation, compensatory mechanisms prevent large fluctuations, with variability potentially rising only in more established hypertensive states.

Measurement Artifacts and Sample Characteristics [33]: Differences in ABPV between the groups could also result from population characteristics or inherent measurement variability within ABPM itself. Factors such as differing stress responses, daily routines, or sample size variations might have impacted the results, leading to an unexpectedly lower ABPV in the high–normal and grade 2 groups.

Increased BPV reflects alterations in functional and structural cardiovascular mechanisms, subclinical or established cardiovascular damage, or underlying pathological conditions, all of which are associated with a poorer prognosis [34]. While there are more data available on office BPV for predicting cardiovascular outcomes compared to home BPV or ABPV, the prognostic value of office BPV depends on the measurement methodology [35]. It is important to note that office BP levels do not capture dynamic changes in BPV induced by daily activities. Home blood pressure monitoring (BPM) requires user training and medical supervision [36].

The independent association of short-term BPV from 24 h Ambulatory Blood Pressure Monitoring (ABPM) recordings with preclinical organ damage is supported by a meta-analysis that found a link between the standard deviation (SD) of 24 h systolic BP (SBP) and daytime SBP with greater Left Ventricular Mass (LVM) [37]. Moreover, meta-analyses have shown that increased short-term BPV from ABPM is associated with a higher risk of cardiovascular events [2]. Our study aligns with these findings, as we observed that 24 h ambulatory SBP variability >14 levels were associated with greater LVM and Left Ventricular Mass Index (LVMI), which is consistent with previous research.

Abramson et al. demonstrated positive associations between markers of inflammation and BPV in healthy, normotensive adults [38]. In newly diagnosed hypertensive individuals, independent of SBP, hs-CRP, and E-selectin levels are related to awake SBPV [39]. Kim et al. observed an association between BPV and inflammatory markers such as TNF-α and IL-6 in hypertensive patients [40]. In a post hoc analysis of a Multi-Ethnic Study of Atherosclerosis (MESA) study, Wong, K. H. et al. found a significant association of IL-6 with higher BPV levels [41]. Ciabano et al. showed that E-selectin, which is an endothelial cell adhesion molecule involved in vascular inflammation, is associated with increased ambulatory diastolic blood pressure variability in patients with type 2 diabetes [42]. Xu, C. et al. suggested that patients with a higher level of hsCRP tended to have larger blood pressure fluctuations [43]. Our study similarly uncovered a significant relationship between inflammation and ABPV, but with the distinction of evaluating 24 h ambulatory SBPV instead of office BPV, and the utilization of SII as a novel marker of inflammation.

To our knowledge, this is the first study to report statistically significant positive associations between SII and 24 h SBPV in newly diagnosed hypertensive patients. Our findings suggest that SII may serve as an independent predictor of increased cardiovascular risk related to ABPV specifically within a relatively healthy, newly diagnosed hypertensive population. Given our selection criteria, these results are likely most relevant for clinicians managing similar patient profiles. The study population’s age and health factors should be considered when extrapolating these findings to other hypertensive populations with broader age ranges or coexisting diseases.

## 6. Limitations

The present study is subject to several limitations. Firstly, due to its cross-sectional design, we are unable to establish causality between Blood Pressure Variability (BPV) and inflammation. To elucidate the causal relationship between BPV and inflammation, future research may require long-term follow-up studies or well-designed intervention studies.

Secondly, the timing of various measurements, including awakening and sleep, relied on self-reported patient information. Utilizing actigraphy or objective measures might offer more precise definitions of these time periods.

These limitations underscore the need for further investigations to comprehensively understand the relationship between BPV and inflammation and to overcome the constraints inherent in the current study design.

## 7. Conclusions

This study demonstrates a significant association between the Systemic Immune Inflammation Index (SII) and Ambulatory Blood Pressure Variability (ABPV) in newly diagnosed hypertensive patients. SII emerged as an independent predictor of ABPV > 14, indicating that elevated inflammation levels correlate with higher BP variability. Moreover, ABPV was associated with increased Left Ventricular Mass (LVM) and Left Ventricular Mass Index (LVMI), underscoring the potential utility of ABPM in identifying patients at heightened cardiovascular risk. SII may serve as a practical inflammatory marker in managing hypertension-related complications, particularly in patients with high ABPV.

Clinicians should interpret these findings as most applicable to newly diagnosed hypertensive patients who meet similar demographic and health criteria as those in our study. Further research could explore whether these findings extend to hypertensive populations with more varied age and health profiles.

## Figures and Tables

**Table 1:** Demographic, echocardiographic, and clinical variables of the study population sample.

	Normal (58)	High–Normal (60)	Grade 1 (53)	Grade 2 (102)	Severe (113)	
	Mean ± Sd	Mean ± Sd	Mean ± Sd	Mean ± Sd	Mean ± Sd	*p* Value (Between the Groups)
SII	412.90 ± 123.6 ^d,e^	425.21 ± 142.5 ^d,e^	442.78 ± 149.9 ^d,e^	746.49 ± 202.6 ^a,b,c,e^	851.44 ± 224.94 ^a,b,c,d^	<0.001
Age (years)	59.71 ± 10.22	54.87 ± 8.92	56.51 ± 11.60	55.52 ± 10.97	55.78 ± 10.41	0.068
Height (cm)	169.03 ± 8.43	171.13 ± 7.30	170.64 ± 8.12	169.18 ± 7.62	167.90 ± 6.32	0.090
Weight (kg)	73.98 ± 11.11	74.53 ± 12.13	75.25 ± 9.39	73.30 ± 10.21	71.64 ± 11.11	0.656
BMİ, kg/m^2^	25.81 ± 2.65	25.42 ± 3.43	25.91 ± 3.22	25.64 ± 3.24	25.39 ± 3.49	0.391
Creatine, mg/dL	0.70 ± 0.14	0.77 ± 0.15	0.75 ± 0.19	0.76 ± 0.16	0.75 ± 0.17	0.122
Gender (female), N (%)	41 (70.7%)	29 (48.3%)	33 (62.3%)	55 (53.9%)	68 (60.2%)	0.115
Glucose, mg/dL	100.09 ± 14.17	97.90 ± 11.00	100.02 ± 11.77	98.18 ± 12.77	95.22 ± 12.44	0.289
Uric acid, mg/dL	4.08 ± 1.37	4.25 ± 1.20	4.48 ± 1.68	4.27 ± 1.25	4.45 ± 1.42	0.415
Cholesterol, mg/dL	201.22 ± 41.77	208.17 ± 40.28	209.79 ± 42.76	211.47 ± 45.37	204.43 ± 44.13	0.990
HDL, mg/dL	59.34 ± 66.58	48.67 ± 10.66	45.32 ± 11.18	45.61 ± 10.79	45.69 ± 10.24	0.019
LDL, mg/dL	120.28 ± 32.44	129.03 ± 38.52	130.81 ± 31.35	132.09 ± 35.59	124.34 ± 35.02	0.163
Triglyceride, mg/dL	158.43 ± 90.57	160.28 ± 89.43	192.02 ± 252.7	176.77 ± 147.1	171.66 ± 79.81	0.133
hs-CRP, mg/L	0.40 ± 0.20	0.36 ± 0.17	0.49 ± 0.40	0.43 ± 0.25	0.49 ± 0.31	0.026
Hb, g/dL	14.00 ± 1.59	14.83 ± 1.22	14.31 ± 1.35	14.37 ± 1.63	14.28 ± 1.62	0.175
WBC count, ×10/μL	7.39 ± 2.23 ^e^	7.86 ± 2.97	7.36 ± 1.77 ^e^	8.18 ± 2.61	8.68 ± 2.04 ^a,c^	0.002
PLT count, ×10/μL	271.12 ± 52.58 ^e^	254.85 ± 63.52 ^e^	245.09 ± 49.81 ^d,e^	288.13 ± 73.27 ^c,e^	331.55 ± 67.63 ^a,b,c,d^	<0.001
Neutrophil count, ×10/μL	3.92 ± 1.33	4.27 ± 1.85	4.14 ± 1.32	5.17 ± 1.93	5.53 ± 1.38	0.099
Lymphocyte count, ×10/μL	2.66 ± 0.95	2.63 ± 1.03	2.39 ± 0.70	2.03 ± 0.75	2.28 ± 0.90	0.062
Monocytes count, ×10/μL	0.57 ± 0.22	0.67 ± 0.27	0.57 ± 0.22	0.66 ± 0.23	0.59 ± 0.19	0.006
ABPV	12.79 ± 2.98 ^e^	12.32 ± 2.54 ^c,d,e^	15.08 ± 4.53 ^b^	14.52 ± 4.78 ^b,e^	17.10 ± 5.22 ^a,b,d^	<0.001
DİPPER, N (%)	41 (70.7%) ^d,e^	46 (76.7%) ^d,e^	35 (66.0%) ^e^	43 (42.2%) ^a,b^	35 (31.0%) ^a,b,c^	<0.001
LVDD	47.41 ± 3.29	48.67 ± 3.27	46.96 ± 3.24	46.93 ± 3.22	47.33 ± 3.53	0.693
LVSD	31.06 ± 3.81	31.18 ± 4.11	30.83 ± 3.57	30.87 ± 4.38	30.93 ± 3.68	0.189
İVS	10.21 ± 1.31	10.78 ± 1.11	10.32 ± 1.42	10.46 ± 1.39	11.80 ± 1.30	0.404
PW	9.58 ± 1.01	10.05 ± 1.02	9.95 ± 1.26	9.90 ± 1.22	10.97 ± 1.12	0.217
LVM	166.95 ± 37.50	185.69 ± 34.09	170.19 ± 41.61	170.64 ± 39.42	200.67 ± 38.37	0.464
LVMİ	90.05 ± 20.11	99.98 ± 22.43	90.71 ± 22.74	92.81 ± 23.02	110.86 ± 23.44	0.677
N/L	1.55 ± 0.47 ^d,e^	1.70 ± 0.50 ^d,e^	1.86 ± 0.70 ^d,e^	2.71 ± 0.86 ^a,b,c^	2.64 ± 0.74 ^a,b,c^	<0.001
P/L	110.74 ± 34.06 ^d,e^	107.08 ± 40.68 ^d,e^	110.01 ± 33.22 ^d,e^	153.53 ± 44.98 ^a,b,c^	159.89 ± 46.20 ^a,b,c^	<0.001

a: significantly different from the normal blood pressure group (*p* < 0.005); b: significantly different from the high–normal blood pressure group (*p* < 0.005); c: significantly different from the grade 1 group (*p* < 0.005); d: significantly different from the grade 2 group (*p* < 0.005); e: significantly different from the severe HT group (*p* < 0.005). (Bonferroni correction was used to counteract the multiple comparisons problem.) SII: Systemic Immune Inflammatory Index; BMI: Body Mass Index; HDL: High-Density Lipoprotein; LDL: Low-Density Lipoprotein; hs-CRP: high sensitive CRP; Hb: hemoglobin; WBC: white blood cell; PLT: platelet; ABPV: Ambulatuary Blood Pressure Variability; LVDD: Left Ventricle Diastolic Diameter; LVSD: Left Ventricle Systolic Diameter; IVS: Inter-Ventricular Septum; PW: Posterior Wall; LVM: Left Ventricle Mass; LVMI: Left Ventricle Mass Index; N/L: neutrophil to lymphocyte ratio; P/L: platelet to lymphocyte ratio.

**Table 2:** Demographic, echocardiographic, and clinical variables of the hypertension groups.

	Group 1 (Non-Candidate for Antihypertensive Therapy) (171)	Group 2 (Candidate for Antihypertensive Therapy) (215)	
	Mean ± Sd	Mean ± Sd	*p*
SII	426.48 ± 138.54	801.65 ± 220.47	<0.001
Gender (female), N (%)	103 (60.2%)	123 (57.2%)	0.550
Age (years)	57.02 ± 10.39	55.66 ± 10.66	0.208
BMİ, kg/m^2^	25.70 ± 3.11	25.51 ± 3.37	0.563
Glucose, mg/dL	99.30 ± 12.36	96.62 ± 12.66	0.038
Creatine, mg/dL	0.74 ± 0.17	0.75 ± 0.16	0.466
Uric acid, mg/dL	4.26 ± 1.42	4.36 ± 1.34	0.475
Cholesterol, mg/dL	206.32 ± 41.49	207.77 ± 44.76	0.743
HDL, mg/dL	51.25 ± 40.00	45.65 ± 10.48	0.050
LDL, mg/dL	126.61 ± 34.49	128.01 ± 35.42	0.697
Triglyceride, mg/dL	169.49 ± 159.05	174.09 ± 116.38	0.743
hs-CRP, mg/L	0.41 ± 0.27	0.46 ± 0.29	0.082
Hb, g/dL	14.39 ± 1.43	14.32 ± 1.62	0.693
WBC count, ×10/μL	7.54 ± 2.39	8.44 ± 2.34	<0.001
PLT count, ×10/μL	257.35 ± 56.57	310.95 ± 73.48	<0.001
Neutrophil count, ×10/μL	4.11 ± 1.53	5.36 ± 1.67	<0.001
Lymphocyte count, ×10/μL	2.57 ± 0.91	2.16 ± 0.84	<0.001
Monocytes count, ×10/μL	0.60 ± 0.24	0.62 ± 0.22	0.323
N/L	1.70 ± 0.57	2.67 ± 0.80	<0.001
P/L	0.02 ± 0.01	0.02 ± 0.01	0.037
ABPV	13.34 ± 3.59	15.87 ± 5.17	<0.001
Dipper (N/%)	122 (71.3%)	78 (36.3%)	<0.001
LVDD, mm	47.71 ± 3.33	47.14 ± 3.38	0.096
LVSD, mm	31.03 ± 3.83	30.90 ± 4.02	0.746
İVS, mm	10.44 ± 1.30	11.16 ± 1.50	<0.001
PW, mm	9.86 ± 1.11	10.46 ± 1.28	<0.001
LV MASS	174.53 ± 38.39	186.43 ± 41.59	0.004
LVMİ	93.74 ± 22.13	102.30 ± 24.89	<0.001

SII: Systemic Immune Inflammatory Index; BMI: Body Mass Index; HDL: High-Density Lipoprotein; LDL: Low-Density Lipoprotein; hs-CRP: high sensitive CRP; Hb: hemoglobin; WBC: white blood cell; PLT: platelet; ABPV: Ambulatuary Blood Pressure Variability; LVDD: Left Ventricle Diastolic Diameter; LVSD: Left Ventricle Systolic Diameter; IVS: Inter-Ventricular Septum; PW: Posterior Wall; LVM: Left Ventricle Mass; LVMI: Left Ventricle Mass Index; N/L: neutrophil to lymphocyte ratio; P/L: platelet to lymphocyte ratio.

**Table 3:** Comparison of demographic, echocardiographic, and clinical variables according to ABPV.

	ABPV < 14 (203)	ABPV > 14 (183)	*p*
	Mean ± Sd.	Mean ± Sd.	
N/L	1.89 ± 0.70	2.61 ± 0.85	<0.001
P/L	122.49 ± 41.53	150.48 ± 50.25	<0.001
LVMİ	96.31 ± 23.35	100.93 ± 24.64	0.060
LV MASS	177.18 ± 38.89	185.56 ± 42.05	0.043
PW, mm	9.99 ± 1.17	10.42 ± 1.28	0.001
IVS, mm	10.64 ± 1.34	11.06 ± 1.54	0.004
LVSD, mm	30.85 ± 3.98	31.07 ± 3.87	0.576
LVDD, mm	47.52 ± 3.24	47.25 ± 3.50	0.431
DİPPER, N (%)	110 (54.2%)	90 (49.2%)	0.327
ABP Grade	2.00 ± 1.42	2.83 ± 1.30	<0.001
SII	510.95 ± 210.65	773.54 ± 251.05	<0.001
Gender (female), N (%)	112 (55.2%)	114 (62.3%)	0.156
Age (years)	56.14 ± 10.27	56.38 ± 10.87	0.828
BMİ, kg/m^2^	25.40 ± 3.20	25.80 ± 3.30	0.232
Glucose, mg/dL	96.49 ± 12.50	99.26 ± 12.52	0.030
Creatine, mg/dL	0.74 ± 0.16	0.74 ± 0.16	0.917
HDL, mg/dL	50.76 ± 36.79	45.20 ± 10.99	0.050
LDL, mg/dL	130.12 ± 34.95	124.36 ± 34.84	0.106
hs-CRP, mg/L	0.40 ± 0.21	0.48 ± 0.33	0.007
Hb, g/dL	14.44 ± 1.42	14.24 ± 1.64	0.211
Triglyceride, mg/dL	161.13 ± 78.23	184.16 ± 180.25	0.111
Cholesterol, mg/dL	209.71 ± 41.66	204.25 ± 44.96	0.218

SII: Systemic Immune Inflammatory Index; BMI: Body Mass Index; HDL: High-Density Lipoprotein; LDL: Low-Density Lipoprotein; hs-CRP: high sensitive CRP; Hb: hemoglobin; WBC: white blood cell; PLT: platelet; ABPV: Ambulatuary Blood Pressure Variability; LVDD: Left Ventricle Diastolic Diameter; LVSD: Left Ventricle Systolic Diameter; IVS: Inter-Ventricular Septum; PW: Posterior Wall; LVM: Left Ventricle Mass; LVMI: Left Ventricle Mass Index; N/L: neutrophil to lymphocyte ratio; P/L: platelet to lymphocyte ratio.

**Table 4:** Linear and multiple linear regression analysis for independent predictors of ABPV.

	Univariate Analysis		Multivariate Analysis		
	r	*p*	Beta	%95 CI	*p*
Age	0.011	0.828	0.001	−0.003 −0.005	0.672
N/L	0.420	0.000	0.074	−0.007 −0.155	0.072
P/L	0.292	0.000	−0.001	−0.002 −0.001	0.198
Gender	−0.073	0.157	−0.059	−0.164 −0.045	0.264
SII	0.495	0.000	0.001	0.001 −0.001	<0.001
hs-CRP	0.141	0.005	0.098	−0.060 −0.255	0.225
BMİ	0.061	0.232	0.008	−0.006 −0.021	0.272
Creatine	0.005	0.917	−0.014	−0.326 −0.299	0.932
LDL	0.082	0.106	−0.001	−0.002 −0.000	0.142
ABP Grade	0.291	0.000	−0.022	−0.064 −0.020	0.295

SII: Systemic Immune Inflammatory Index; BMI: Body Mass Index; hs-CRP: high sensitive CRP; N/L: neutrophil to lymphocyte ratio; P/L: platelet to lymphocyte ratio.

## Data Availability

The original contributions presented in the study are included in the article; further inquiries can be directed to the corresponding author(s).
